# Treating older patients with AIDS using Traditional Chinese Medicine Combined with Conventional Western Medicine in China

**DOI:** 10.14336/AD.2021.0925

**Published:** 2021-12-01

**Authors:** Zhibin Liu

**Affiliations:** ^1^Department of AIDS Treatment and Research Center, the First Affiliated Hospital of Henan University of Chinese Medicine, Zhengzhou, China.; ^2^Henan Key Laboratory of Viral Diseases Prevention and Treatment of Traditional Chinese Medicine, The First Affiliated Hospital of Henan University of Chinese Medicine, Zhengzhou, China

**Keywords:** AIDS, combination anti-retroviral therapy (cART), Traditional Chinese medicine, Western medicine, mortality, immune reconstitution

## Abstract

Acquired immunodeficiency syndrome (AIDS) is a chronic and incurable disease. People with human immunodeficiency virus (HIV) require lifelong care. Traditional Chinese Medicine (TCM) has played an important role in AIDS treatment since the year 2004. TCM offers many advantages including a rich resource of Chinese herbs, lower cost and fewer side effects. In addition to the widespread use of antiviral therapy, TCM offers unique humanistic care and holistic adjustment of the body system. To date, more and more patients are benefiting from TCM not only in China. In this article, we describe the feasibility of treating AIDS with TCM.

Being a chronic and controllable infectious disease, treatment and research related to human immune-deficiency virus (HIV) or acquired immunodeficiency syndrome (AIDS) has been ongoing for more than 30 years [1,2]. China is a low-prevalence country with nearly 85,000 people living with HIV at the end of 2018 [3]. The mortality of patients with AIDS has been considerably reduced since the advent of antiviral therapy, and life expectancy has increased dramatically, nearly reaching that of the general population. However, new HIV infections among older people are on the rise. As a chronic and incurable disease, lifelong medical care is required for survival among patients living with HIV. Antiviral therapy is often limited by drug side effects, adherence to therapy, social discrimination, and even high drug resistance^[^4^]^. In developing countries with limited medical resources and a lack of health knowledge among the population, most patients with AIDS live in poor areas with limited opportunities to receive Western medical care, other than free antiviral therapy.

In clinical practice, Traditional Chinese Medicine (TCM) has a theoretical basis and a profound cultural heritage, with a rich accumulation of clinical experience. Under the guidance of TCM theory, Chinese medicine practitioners have used TCM to successfully treat numerous infectious diseases, such as severe acute respiratory syndrome, influenza A virus sub-type H1N1, epidemic encephalitis, hepatitis, and COVID-19 [5]. TCM and conventional western medicine (WM) are both considered mainstream medicine, with the support and promotion of the government of China, where medical care is not limited to WM.

There are a large number of TCM hospitals and clinics in China, where millions of patients receive TCM treatment and services every day. TCM involves a variety of approaches including qigong, Chinese herbs, acupuncture, and moxibustion. Patients often seek treatment with TCM alone or in combination with WM to manage HIV/AIDS, and this conventional practice has flourished in China. The unrecognized efficacy of TCM in the treatment of AIDS might be owing to fundamental differences in the two kinds of medicine. The gold standard diagnostic criteria of the World Health Organization (WHO) for AIDS are CD4^+^ T-cell count and HIV viral load. TCM is more concerned with the clinical complaints of patients with AIDS, including abnormal symptoms and signs, lower level of quality of life (QOL), complications, opportunistic infections, and side effects of combination anti-retroviral therapy (cART) and not merely CD4^+^ T cell counts and viral lead-time is based on clinical practice with humanistic characteristics, a holistic approach, syndrome differentiation, and flexible prescription, which lead many Chinese medicine practitioners and patients to believe that TCM can treat any complex disease.

TCM treatment of AIDS is a process of continuous improvement. Since 2004, the State Administration of Traditional Chinese Medicine of China has executed a pilot program for the use of TCM to treat patients with AIDS in conjunction with anti-retroviral drugs and has sponsored many AIDS-related science and technology projects [6], and with several national projects funded by the Chinese government, the various modalities for the treatment and management of AIDS using TCM have been developed [7]. As a result, the role of TCM in the treatment of AIDS has been established.

## Multiple effects of TCM in AIDS treatment

TCM has multiple effects [8], such as ameliorating symptoms and signs, increasing CD4^+^ T cell counts, reducing plasma HIV viral loads, reducing opportunistic infections, improving QOL, counteracting the effects of anti-retroviral drugs, promoting immune function recovery, and increasing survival of patients with AIDS. TCM offers many advantages including a rich resource of Chinese herbs, lower cost, and fewer side effects.

## Alleviating signs and symptoms

Signs and symptoms (e.g., diarrhea, fatigue, insomnia, weakness, weight loss) are more common among patients with AIDS who have lower immune function [9], which may lower the patient's QOL. Chinese medicine practitioners often use syndrome differentiation or a fixed formula to treat AIDS, effectively alleviating some of the signs and symptoms.

A multi-center, randomized, double-blind, controlled clinical trial demonstrated the effects of Immune No. 2 granules on signs and symptoms of patients with AIDS who had low viral loads and low immune status after cART [10]. A total of 384 participants were randomized into the TCM group (192 receiving Immune No.2 granules+cART) and the control group (192 receiving placebo+cART). Scores of symptoms and signs in the two groups were analyzed. After 72 weeks, there was no significant difference in the overall scores for symptoms (TCM group 1.09±1.60 vs. control group 0.92±1.52). However, total scores for signs and symptoms (before 2.62±3.07 *vs.* after 1.09±1.60) and scores for fatigue and anorexia in the TCM group were significantly lower than those in the control group after treatment (fatigue: 0.41±0.89 vs. 0.59±0.92 and anorexia: 0.13±0.49 *vs.* 0.45±0.84). The study findings suggest that Immune No. 2 granules have a certain therapeutic effect on fatigue and anorexia symptoms in patients with AIDS who have a low viral load and low immune status after cART.

Meng *et al.* [11] reported a random control study to test the effects of Gancao Xiexin decoction on the symptoms, signs, and QOL of patients with AIDS who had recurrent oral ulcers owing to dampness-heat syndrome of the spleen and stomach. A total of 78 patients with AIDS were randomized into the TCM treatment group (52 patients) and WM group (26 patients). The results showed that Gancao Xiexin decoction could improve patients’ signs and symptoms. As for QOL, the TCM group had better improvement than the WM group on day 14, but there was no significant difference. The study results suggest that Gancao Xiexin decoction can improve signs and symptoms in patients with AIDS who have recurrent oral ulcers.

Ma *et al.* [12] randomized 141 patients with AIDS and pulmonary infection who had syndrome of phlegm-heat obstructing the lung into a group given Qingfei Peiyuan Micro-pill (94 patients) and a group treated with WM (47 patients). All patients were treated routinely. The authors observed obvious improvement in patients’ signs and symptoms and QOL among patients in the TCM group. The trial suggested that Qingfei Peiyuan Micro-pill helps to alleviate the signs and symptoms and improves QOL in patients with AIDS who have pulmonary infection.

Xu *et al.* [13] reported the efficacy of a Chinese herbal formulation (Xielikang capsule) in AIDS-related chronic diarrhea in a randomized, double-blind, double dummy, controlled clinical trial. A total of 156 patients were randomized into a group that received Xielikang capsule and a group receiving loperamide. Patients in the TCM group were administered Xielikang capsule and loperamide placebo and patients in the loperamide group were administered loperamide and Xielikang capsule placebo, each for 14 days. The results showed that Xielikang capsule could more significantly reduce stool weight and diarrhea questionnaire scores than loperamide, with no significant differences in stool frequency.

## Improving QOL

QOL is an individual's perception of their life in the context of the culture and value systems in which they live and in relation to their goals, expectations, standards and concerns [14]. The QOL of patients with HIV/AIDS are significantly lower than that of the general population [15]. Some research has focused on improving the QOL of patients using TCM methods, which offer great advantages in improving patients' QOL.

Jin *et al.* [16] reported a cross-sectional investigation to explore the effects of TCM on the life quality of older patients with AIDS in a provincial TCM project in Henan. A total of 410 older patients with AIDS were ultimately included, 220 who were treated with TCM and 190 treated with a non-TCM approach. The results of multiple linear regression showed that the life quality of patients treated with TCM was superior to that of their non-TCM counterparts. The study findings suggest that TCM can improve QOL in older patients with AIDS.

Weng *et al*. [17] reported a randomized, single-center, parallel control study to test a Chinese herbal formulation (Qingdu capsule) in 80 older patients with HIV/AIDS. Patient QOL was evaluated and CD4 cell counts, and viral loads were measured at baseline and at 3-, 6-, and 12-months during follow-up. The results showed that Qingdu capsule combined with cART could improve immune function, maintain the virus inhibition rate, and improve patients’ QOL and medication compliance.

Xu *et al.* [18] reported a prospective study examining QOL among asymptomatic HIV-infected patients in a randomized, double-blind, placebo parallel control study. In total, 1200 patients in the asymptomatic phase of HIV infection were enrolled in 18-month follow-up. The WHOQOL-HIV scale was used in the trial. The study results showed that QOL in the TCM group improved more than that in the placebo group, which suggests that TCM could significantly improve patients’ QOL.

## Benefiting long-term survival

AIDS remains a highly fatal disease. Survival rates reflect the lethality of the disease and can be used to evaluate the long-term curative effect of AIDS treatments.

Zhao *et al.* [19] reported the 8-year survival of patients with AIDS who were treated with TCM. The study included 385 patients with AIDS, 165 of whom used a 16-herb formula (i.e. TCM) for 14 days to 9 months. The results revealed an 8-year survival rate of 87% for TCM users and 34% for non-TCM users. No deaths occurred in patients who used TCM for 6 to 9 months, and none of the survivors experienced a relapse of AIDS or any severe adverse events. This study suggested that TCM might be beneficial for long-term survival in this patient population.

In a 6-year retrospective cohort study [20], 1,666 patients with HIV/AIDS treated with TCM were included. A total of 312 (18.7%) patients died within 6 years. The total mortality rate over the study period was 3.6 per 100 person-years, which was lower than the worldwide rate. This study indicates that TCM might increase survival and lengthen the life span in patients with HIV/AIDS.

Xu *et al.* [21] reported a retrospective cohort study including 528 patients with AIDS, with 322 in the TCM+cART group and 206 in the cART group. After 8 years, the mortality in the TCM+cART group was 3.3/100 person-years, which was lower than that in the cART group of 5.3/100 person-years. The hazard ratio for mortality in the cART group was 1.6 times that of the TCM+cART group. According to the study results, TCM might help to prolong life and decrease mortality in patients with AIDS.

## Reducing side effects of antiviral drugs

The morbidity and mortality of AIDS has been reduced since the introduction of cART, but adverse effects of these drugs remain inevitable. Antiviral strategies are limited in developing countries because patients must adhere to treatment for their entire life. It very important to seek more realistic methods to reduce the side effects of cART drugs.

A multi-center, randomized, double-blind, placebo controlled clinical trial evaluated the efficacy and safety of TCM in reducing cART adverse reactions [22]. 536 patients were administered TCM (268 patients received Aikeqing or Aifukang capsule+cART) or placebo (268 received Aikeqing placebo or Aifukang placebo+cART). During the observation period, Aikeqing and Aifukang capsule or placebo were used according to syndrome differentiation. After 72 weeks, the rate and severity of adverse reactions in the treatment group were lower than those in the control group, and medication compliance was higher in the TCM group. This research proved that cART combined with TCM can reduce the incidence and severity of adverse drug reactions.

Wendan granule is composed of eight Chinese herbs to alleviate adverse gastrointestinal effects caused by cART [23]. The incidence of gastrointestinal reactions to cART in the group that received Wendan granule was 20.4%, which was lower than the 48.0% in the placebo group. The symptoms score for gastrointestinal adverse effects was significantly different between the two groups, as were scores before and after treatment. This study suggests that Wendan granule can reduce the incidence of adverse gastrointestinal symptoms caused by cART, especially mild or moderate symptoms.

Jiang *et al.* [24] observed the effect of Jingyuankang capsule, a Chinese herbal formulation, on leukocyte levels in patients with AIDS in a randomized double-blind trial. After 6 weeks of administration, Jingyuankang capsule had a better therapeutic effect in treating peripheral leukopenia than the effect in the control group.

Danggui Shaoyao San, a well-known Chinese herbal formula, has been used to treat cART-induced liver dysfunction [25]. Studies have shown that this preparation can reduce cART-induced liver dysfunction by lowering elevated liver enzyme levels and can alleviate symptoms like abdominal distension, diarrhea, and hypochondriac pain.

## Promoting immune reconstitution

Some individuals exhibit failed or incomplete recovery of CD4+ cell counts despite viral load suppression after continuous cART. This phenomenon is defined as suboptimal immune recovery or poor immune reconstitution (IR) [26]. IR is defined as recovery to normal or near normal levels after cART or TCM. After achieving IR, the incidence of AIDS-related opportunistic infections and tumors is decreased, and patient mortality and morbidity are reduced.

TCM has been used to promote IR and some studies have proven that TCM has an important role in IR. A randomized, double-blind, placebo-controlled clinical trial tested a Chinese herbal formulation (Immune No. 2) with respect to IR in patients with HIV/AIDS after cART [27]. The study showed that Immune No. 2 could effectively improve CD4, CD45RA, and CD45RO counts, thereby promoting IR.

Another randomized, double-blind, placebo parallel controlled clinical trial investigated a Chinese herbal formulation (Zhongyan-4) in treating patients with HIV/AIDS in the early or middle stages [28]. The trial suggested that the efficacy of Zhongyan-4 is superior to that of placebo in elevating CD4^+^, CD45RA^+^, and CD8^+^ T-cell counts, reducing HIV virus load, improving clinical symptoms and signs, and increasing body weight in patients.

## Improving laboratory results

The indexes viral load and CD4^+^ T-cell count have been adopted in TCM. Some results demonstrate that TCM shows promise in reducing HIV viral loads, increasing CD4^+^ T cell counts, as well as improving other immunological indicators. Compared with HIV viral loads, CD4^+^ T cell counts are more frequently examined in TCM clinical trials.

Sun *et al.* [29] reported results of a retrospective cohort study including 2954 patients with HIV/AIDS. Patients were divided into three groups according to their recorded treatment regimens. All three regimens improved patients' CD4^+^ cell counts. Compared with the ART group during the first 6 months, CD4^+^ T cell counts among patients in the TCM group and TCM combined with ART group exhibited a steady rise. CD4^+^ T cell counts in the TCM, and combined groups remained much lower than those in the ART group during the first 3 years, but these overtook the ART group in the fourth year.

A retrospective cohort study analyzed the efficacy of TCM therapy on long-term trends in CD4^+^ T cell counts among patients with HIV/AIDS who were treated with cART over a 14-year period [30]. The study included 307 patients who received TCM+cART and 721 patients treated with cART alone. Among patients in the TCM+cART group with a baseline CD4+T cell count <350 cells/mL, the values tended to increase more rapidly to approximately 350 cells/mL than patients in the cART group. This indicates that TCM can increase the CD4+T cell count among patients with HIV/AIDS who have a baseline CD4 count of <350 cells/mL.

Li *et al.* [31] observed the short- and long-term clinical efficacy of Aike Qing granule (a pure Chinese herbal preparation) in the treatment HIV-infected individuals. According to different treatment methods, 85 patients were divided into a Aike Qing group, cART group, and Aike Qing+cART group. The authors found that CD4+T cell count in the Aike Qing+cART group were significant increased after 24 months as compared with baseline values (520±48.30 cells/mL vs. 407.71±41.20 cells/mL). The study reported that long-term use of Aike Qing granule can stabilize and increase patients’ CD4+T cell counts, improve QOL, and reduce adverse reactions to cART.

The efficacy of a Chinese herbal formulation (Xiang A1 capsule) in AIDS has been demonstrated in a randomized, double-blind, double dummy, controlled clinical trial [32]. Sixty enrolled patients were divided equally into three groups, those receiving Xiang A1 granules+cART placebo, cART+Xiang A1 granules placebo, or Xiang A1 granules+cART. After a 6-month intervention, symptoms and QOL were significantly improved in the three groups. However, the effects were best in the combination group, with significant differences between the Xiang A1 and cART groups. Viral loads were reduced in the cART and combination groups, with no change in the Xiang A1 group. In the Xiang A1 and Xiang A1+cART groups, the percentage of CD45RA+, CD45RA+CD3+, CD45RA+CD28+, CD45RA+CD4+, and D45RA+CD25+, and the ratio of CD4+T to CD8+T cell were all significantly increased. The percentage of CD45RA+, CD45RA+CD3+, and CD45RA+CD4+, and ratio of CD4 to CD8 were significantly increased in the cART group. There was a significant difference between the Xiang A1 group or combination group versus the cART group. These research findings suggest that Xiang A1 granules could improve signs and symptoms in patients with AIDS and excessive dampness owing to spleen deficiency, and there is a synergistic reaction of Xiang A1 with cART. Antiviral effects of Xiang A1 granule are not obvious, but this TCM preparation has obvious effects on IR.

Song *et al.* [33] observed the effects of cART combined with Jianpi Yishen decoction on Chinese medical syndrome score and CD4^+^ T cell counts among patients with AIDS in the asymptomatic stage. Sixty patients were randomly divided into a control group (cART) and treatment group (Jianpi Yishen decoction+cART). After 12 months, the Chinese medical syndrome score decreased and CD4^+^ T cell counts increased significantly in both groups; however, these changes were greater in the TCM+cART group than in the cART group. Thus, the combination of cART and Jianpi Yishen decoction may improve symptoms in asymptomatic patients with AIDS and increase CD4^+^ T cell counts.

Jiang *et al.* [34] reported a cohort study including 34 patients with HIV/AIDS that examined the effects of Fuzheng Paidu granule on regulating immune activation molecules CD38 and human leukocyte antigen-DR (HLA-DR) expressed on CD4^+^ and CD8^+^ T cells. They found that patients with HIV/AIDS exhibited an improved immune activation profile following Fuzheng Paidu granule treatment. The potential mechanism of action for Fuzheng Paidu granule appears to lie in its ability to up-regulate CD38 and HLA-DR expression on CD4^+^ T cells, and down-regulate these on CD8^+^ T cells, thereby modulating immune activation of CD4^+^ and CD8^+^ T cells.

Wang *et al.* [35] reported a randomized, double-blind, placebo-controlled clinical trial testing Aining granule+cART. Although CD4^+^ T-cell counts decreased in both groups after 11 months, cell counts declined less significantly in the Aining group than in the control group. Significant improvement of symptoms such as fatigue, anorexia, nausea, diarrhea, and skin rash were observed in the Aining granule group. However, there was no significant difference between the two groups in viral load after treatment.

## Safety in treating patients

The safe medication use is a basic principle consistently followed by TCM specialists. Whether TCM is safe remains controversial. Only few studies have reported some mild side effects of TCM, indicating that it is a relatively safe treatment option [37]. Treatment with TCM must nevertheless be carried out carefully [36], for some researchers report that certain herbal medicines can interfere with antiviral drug metabolism and can change the concentration of antiviral drugs [38].

## Facilitators of AIDS treatment with TCM

Treating AIDS using TCM is decided not only according to the needs of the patient but also according to the availability and side effects of antiviral drugs. In some regions of China, individuals can easily receive TCM treatment by consulting with Chinese medicine practitioners and via other approaches. First, the national TCM project is the main mode for receiving TCM treatment. The State Administration of Traditional Chinese Medicine of China has carried out a trial program, with different fixed preparations and additional TCM treatments supplied to participants in five different provinces of China. Second, the free clinic model is very convenient. Patients usually do not have to inform Chinese medicine practitioners of their HIV status at an ordinary TCM clinic when they see a doctor, and the CMP usually makes a diagnosis and treats according to the patient's clinical information using a TCM approach. Third, the self-management model of TCM is popular. Individuals can easily receive TCM treatment or health care whenever they wish. Using over-the-counter herbal medicines and preparations for tea and medicinal meals as well as non-drug therapy (qigong, massage, acupuncture) are popular modes of health management in TCM.

## Conclusions

TCM has an important role in AIDS treatment, offering unique humanistic care and holistic adjustment of the body system. The use of TCM has become more common among people living with HIV, and increasingly more patients with AIDS are benefiting from TCM. Some research has shown encouraging results, and numerous evidence-based high-quality clinical studies have confirmed the effectiveness and safety of a TCM approach in managing HIV, alone or in combination with WM. Previous studies have mainly focused on younger patients with AIDS, with few studies among older patients. Therefore, active promotion and further high-level evidence in support of TCM treatment for HIV/AIDS are needed.
